# Exploring the Use of Social Media for Medical Problem Solving by Analyzing the Subreddit r/medical_advice: Quantitative Analysis

**DOI:** 10.2196/56116

**Published:** 2025-03-20

**Authors:** Xiyu Zhao, Victor Yang, Arjun Menta, Jacob Blum, Padmini Ranasinghe

**Affiliations:** 1 School of Medicine The Johns Hopkins University Baltimore, MD United States

**Keywords:** online health information, medical advice, Reddit, r/medical_advice, health information–seeking behavior, user-generated content, subreddits, patient education, virtual environments, information quality, social media, medical problem, quantitative analyses, cross-sectional study, user interactions, online health, decision-making, social news, health information

## Abstract

**Background:**

The advent of the internet has transformed the landscape of health information acquisition and sharing. Reddit has become a hub for such activities, such as the subreddit r/medical_advice, affecting patients’ knowledge and decision-making. While the popularity of these platforms is recognized, research into the interactions and content within these communities remains sparse. Understanding the dynamics of these platforms is crucial for improving online health information quality.

**Objective:**

This study aims to quantitatively analyze the subreddit r/medical_advice to characterize the medical questions posed and the demographics of individuals providing answers. Insights into the subreddit’s user engagement, information-seeking behavior, and the quality of shared information will contribute to the existing body of literature on health information seeking in the digital era.

**Methods:**

A cross-sectional study was conducted, examining all posts and top comments from r/medical_advice since its creation on October 1, 2011. Data were collected on March 2, 2023, from pushhift.io, and the analysis included post and author flairs, scores, and engagement metrics. Statistical analyses were performed using RStudio and GraphPad Prism 9.0.

**Results:**

From October 2011 to March 2023, a total of 201,680 posts and 721,882 comments were analyzed. After excluding autogenerated posts and comments, 194,678 posts and 528,383 comments remained for analysis. A total of 41% (77,529/194,678) of posts had no user flairs, while only 0.1% (108/194,678) of posts were made by verified medical professionals. The average engagement per post was a score of 2 (SD 7.03) and 3.32 (SD 4.89) comments. In period 2, urgent questions and those with level-10 pain reported higher engagement, with significant differences in scores and comments based on flair type (*P*<.001). Period 3 saw the highest engagement in posts related to pregnancy and the lowest in posts about bones, joints, or ligaments. Media inclusion significantly increased engagement, with video posts receiving the highest interaction (*P*<.001).

**Conclusions:**

The study reveals a significant engagement with r/medical_advice, with user interactions influenced by the type of query and the inclusion of visual media. High engagement with posts about pregnancy and urgent medical queries reflects a focused public interest and the subreddit’s role as a preliminary health information resource. The predominance of nonverified medical professionals providing information highlights a shift toward community-based knowledge exchange, though it raises questions about the reliability of the information. Future research should explore cross-platform behaviors and the impact of misinformation on public health. Effective moderation and the involvement of verified medical professionals are recommended to enhance the subreddit’s role as a reliable health information resource.

## Introduction

The internet has significantly impacted how individuals access and share health-related information. Online health information–seeking behavior has been a growing area of interest in the medical literature, given its potential impact on patient knowledge, decision-making, and outcomes [1]. As a result, the quality and accuracy of health information shared on the internet have been the subject of numerous studies, which have identified both benefits and risks for users [2,3].

Reddit, a social news forum and discussion website, has emerged as a popular platform for health information sharing [4]. Among its topic-specific communities called “subreddits,” r/medical_advice has become a prominent online community where users seek and provide advice related to medical conditions, symptoms, and treatments [5]. r/medical_advice stands out not only for its popularity but also for its extensive user engagement compared with other similar online communities. Despite its popularity, there has been limited research examining the content and user interactions within this online community [6]. As the demand for patient education in internet-based environments continues to grow, it is essential to understand the topics discussed on this subreddit to assess the quality of the information provided, as well as the challenges associated with providing accurate and reliable health information in online spaces.

We define information-seeking behavior as the deliberate pursuit of health-related knowledge by individuals, which differs from information sharing (actively providing knowledge to others) and more general health communication (exchanging health-related messages with various purposes). By focusing on r/medical_advice, we specifically examine users seeking preliminary guidance or reassurance before consulting health care professionals. This study addresses three main research questions: (1) What types of medical questions are asked on r/medical_advice? (2) How do different post flairs, pain levels, and inclusion of media relate to user engagement? and (3) How do verified and nonverified medical professionals contribute to the information ecology of r/medical_advice? The findings of this study will contribute to the growing body of literature on health information–seeking behavior in the digital age and help inform potential strategies for improving the quality and utility of online health information.

## Methods

### Study Design and Data Collection

This cross-sectional study systematically characterized all posts and their top comments from the r/medical_advice subreddit since its inception on October 1, 2011. Data for this investigation were collected on March 2, 2023, using a public resource created by Jason Baumgartner of pushshift.io [7]. Metadata fields collected for posts included subreddit, post ID, title, self-text, post flair, comment score, post score, author, author flair, URL, image, time stamp, and date (Table 1). Flairs are a feature that allows users to add a label or tag to their posts or usernames. Post flairs categorize post content, while user flairs (also referred to as author flairs) can indicate qualifications or expertise in a specific subject. For comments, the collected metadata fields included subreddit, comment content, score, author, author flair, post ID, URL, image, time stamp, and date. Before analysis, we applied data cleaning steps to remove non–user-generated content and posts that did not represent genuine user inquiries such as automated moderation posts, duplicate entries, or advertisements. We used similar criteria for comments to ensure that both posts and comments represented organic user activity.

**Table 1:** Definition of metadata fields. This table provides definitions for the common metadata fields encountered in the pushshift.io database.

Metadata field	Definition
Subreddit	The name of the specific Reddit community where the post is made
Post ID	A unique identifier assigned to each post in a subreddit
Title	The heading or title of the Reddit post
Self-text	The main body text of the Reddit post
Post flair	A category or tag assigned to a post to indicate its content or topic
Comment score	A numerical value representing the net upvotes and downvotes a comment receives
Post score	A numerical value representing the net upvotes and downvotes a post receives
Author	The username of the individual who created the post
Author flair	A tag or label next to a user's name that indicates their role, expertise, or affiliation
URL	A direct link to the specific Reddit post
Image	Visual content (photo or graphic) attached to a Reddit post
Time stamp	The exact date and time when the post or comment was made
Date	The date when the post was made, formatted as year-month-day

### Subreddit Time Periods and Flair Analysis

The analysis of posts was divided into 3 distinct time periods: October 1, 2011, to March 5, 2019 (period 1); March 6, 2019, to July 31, 2022 (period 2); and August 1, 2022, to March 2, 2023 (period 3). This categorization was necessary due to the varying availability of flairs during these periods. Period 1 had no available flairs, whereas period 2 offered flair options based on pain level or question type. In period 3, flairs were organized using a systems-based approach.

The analysis of author flairs was conducted between May 7, 2019, and March 2, 2023, which corresponds to the implementation of author flairs. Throughout the entire time period, user flair options remained consistent. Flairs related to each post, the account that submitted the post, and comments were analyzed.

### Definition of Scores

Scores were defined as the net result of upvotes subtracted by downvotes, with a lower limit set at 0. Upvotes and downvotes on Reddit signify agreement, relevance, or perceived quality of a post or comment. A higher score typically indicates greater community acceptance.

### Statistical Analysis and Data Visualization

RStudio (Posit) was used for all statistical analyses, while data visualization was conducted using GraphPad Prism 9.0 (Insight Partners).

### Data Analysis

The data analysis process involved the calculation of averages and SDs for posts across the 3 time periods. To comprehensively examine the engagement of the subreddit community with the posts, the study considered several factors, including post flair; the presence of images, galleries (multiple images), or videos; and the combined engagement, which was defined as the sum of scores and comments.

A detailed examination of post flair engagement was conducted, comparing engagement across flairs in periods 2 and 3. The Kruskal-Wallis test was initially applied to assess differences in combined engagement, followed by the Dunn test for pairwise comparisons. During period 2, the analysis was segregated into question type (general, urgent, or other) and pain level (no pain, 1-3, 4-6, 7-9, and 10). Since each post could only be assigned a single flair, posts were exclusively categorized based on either question type or pain level.

In period 3, a similar statistical approach was used to compare combined engagement by the type of medical problem. The analysis in this period focused on the systems-based categorization of post flairs, enabling a more targeted investigation of engagement patterns.

### Ethical Considerations

Project data were collected from a publicly accessible online forum. No direct interaction with users occurred, and no personally identifiable information was included in the dataset. In accordance with ethical guidelines for internet research, efforts were made to ensure privacy and confidentiality by excluding usernames and any personally identifiable content from the analysis. The use of Reddit data complies with the platform’s terms of service, which allow the analysis of public content for research purposes. Institutional review board approval was not required, as this study exclusively analyzed publicly available, anonymized data and did not involve human participant interventions.

## Results

### Demographics and Flair Distribution

**Figure 1:**
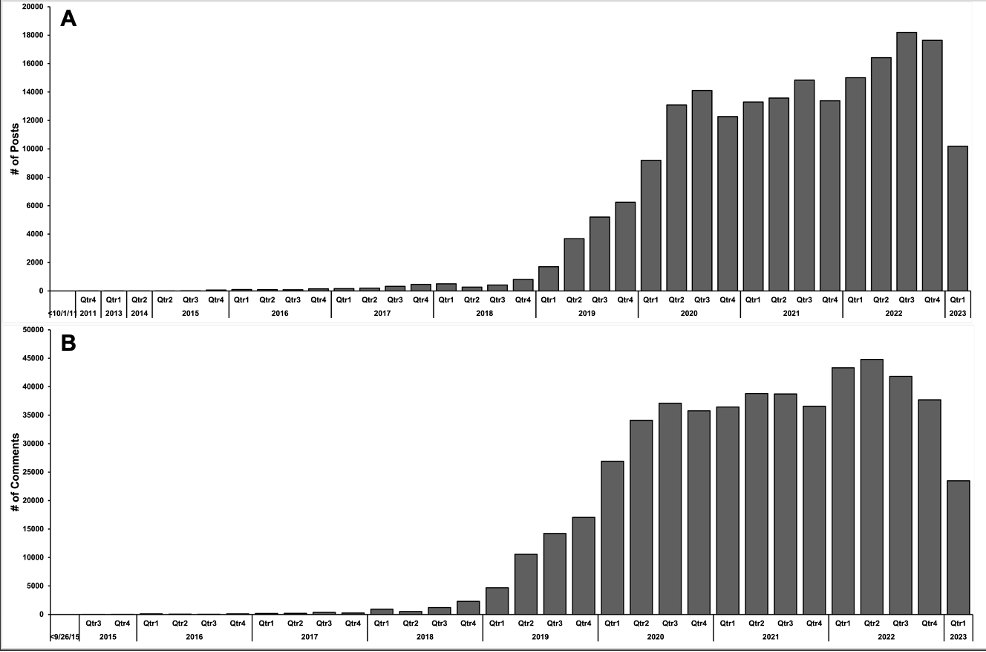
Quarterly trends in posts and comments in r/medical_advice. This bar graph displays the (A) number of posts and (B) comments in the r/medical_advice subreddit over time, with each bar representing a quarter of a year on the x-axis. Qtr: quarter.

A total of 201,680 posts (Figure 1A) and 721,882 comments (Figure 1B) were collected from October 2011, the inception of the subreddit, through March 2023. After data cleaning to remove nonmedical inquiries and responses, 194,678 posts and 528,383 comments remained for analysis. The top flairs of periods 2 and 3 are shown in Tables 2 and 3, respectively.

**Table 2:** Distribution of post flairs in period 2. The table shows the frequency and percentage of post flairs categorized by question type and pain level, illustrating the prevalence of various types of medical questions and reported pain levels in the subreddit during this period.

Post flair type			Post flairs (n=136,486), n (%)
**Question type**			
	General question	50,671 (37.4)	
	Urgent question	17,739 (13.1)	
	Other question	6612 (4.9)	
**Pain level**			
	No pain	23,844 (17.6)	
	Levels 1-3	18,337 (13.5)	
	Levels 4-6	12,252 (9)	
	Levels 7-9	6055 (4.5)	
	Level 10	976 (0.7)	

**Table 3:** Distribution of post flairs in period 3. This table presents the frequency and percentage of post flairs across different medical topics during period 3, highlighting the most discussed health issues in the subreddit during this period.

Post flair	Values (n=27,661), n (%)
Skin issues or rashes or freckles or moles	6505 (23.5)
Mouth or gums or throat or cheeks	2648 (9.6)
Genitalia	2486 (9)
Injury	2447 (8.8)
Bones or joints or ligaments	2198 (7.9)
Digestion or stomach or bowels	2179 (7.9)
Illness	2033 (7.3)
Wound care	2015 (7.3)
Medication	1758 (6.4)
Cardiac	1230 (4.4)
Eyes	903 (3.3)
Mental health	741 (2.7)
Parasite concern	264 (1)
Pregnancy	254 (0.9)

### User Flair Analysis

Across all time periods, 41% (77,529/194,678) of posts were made by users without user flairs, 42% (81,607/194,678) of posts were made by users who were not verified medical professionals, 18% (35,434/194,678) of posts were made by users who were not verified, and 0.1% (108/194,678) of posts were made by verified medical professionals. The verification process on the subreddit requires the user to upload a picture of their employment badge next to their handwritten username. In other words, 99.9% (194,886/194,691) of the posts were made by Redditors who were not verified medical professionals.

With respect to comments across all three periods, 50% (232,274/528,383) of the comments were made by users tagged “Not a Verified Medical Professional,” 39% (183,470/528,383) of the comments were made by users tagged “Users Not Verified,” and 12% (55,296/528,383) of the comments were made by medical professionals. Table 4 illustrates the breakdown of medical professionals by profession.

**Table 4:** Breakdown of medical professionals in comments.

Medical profession	Values (n=55,296), n (%)
Nurses^a^	29,838 (54)
Physicians	11,204 (20.3)
Students^b^	6615 (12)
Emergency medical services personnel^c^	1496 (2.7)
Allied health professionals^d^	962 (1.7)
Medical assistants	468 (0.8)
Midlevel providers^e^	215 (0.4)
Nursing support staff^f^	89 (0.2)
Other (moderators, etc)	4409 (8)

^a^Nurses encompass registered nurses, licensed practical nurses, and licensed vocational nurses.

^b^Students involve medical, nursing, and allied health students.

^c^Emergency medical services personnel consist of paramedics and emergency medical technicians.

^d^Allied health professionals include roles such as respiratory therapists, occupational therapists, physical therapists, and radiologic technologists.

^e^Midlevel providers include nurse practitioners and physician assistants.

^f^Nursing support staff includes certified nursing assistants.

### Engagement Analysis

Across all posts and time periods on the subreddit, on average, each post received a score of 2 (SD 7.03; range 0-687) and 3.32 (SD 4.89; range: 0-338) comments. To account for the total engagement level of the subreddit over time, the following averages were calculated for each period: (1) score of 1.38 (SD 0.98) and 2.17 (SD 2.87) comments in period 1; (2) score of 2.14 (SD 7.71) and 3.56 (SD 5.05) comments in period 2; and (3) score of 1.48 (SD 3.83) and 2.50 (SD 4.30) comments in period 3.

**Figure 2:**
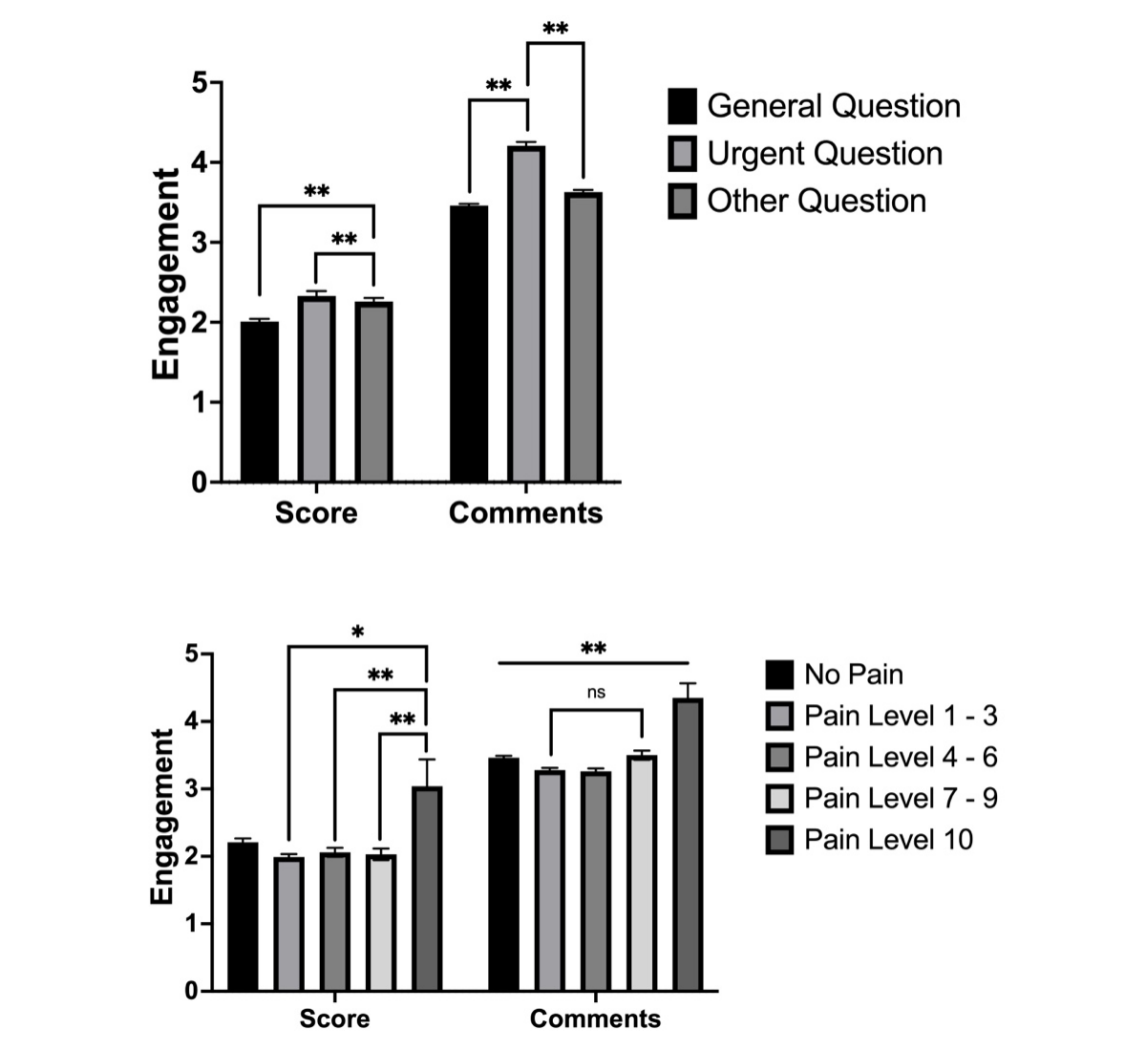
Engagement analysis by question type and pain level in period 2. This figure presents two separate bar graphs, illustrating the engagement patterns in r/medical_advice during period 2. The top graph displays the engagement by question type, including general question, urgent question, and other question, while the bottom graph shows the engagement by pain level categories (no pain, levels 1-3, levels 4-6, levels 7-9, and level 10). Error bars represent the SEM. Asterisks indicate the level of statistical significance (*P<.05 and **P<.01), with all comparisons in the bottom graph being significant except for the one marked as nonsignificant. These graphs highlight the differences in engagement across various question types and pain levels, shedding light on the patterns of user interaction in the subreddit during period 2. ns: nonsignificant.

In period 3, posts were divided into system-based flairs. A total of 11,772 posts were removed from the analysis due to the lack of problem-based flairs, leaving 27,661 posts for flair analysis. Engagement, broken down by flair for period 3 is highlighted, is given in Table 5. Posts related to pregnancy had the highest engagement in period 3, while those about bones, joints, or ligaments had the lowest engagement (Figure 2). This pattern was reflected when examining both scores and comments.

**Table 5:** Engagement by post flair in period 3. This table presents the mean (SD) values of combined engagement (score and comments) for each post flair category during period 3, highlighting the varying levels of engagement across different medical topics within the r/medical_advice subreddit.

Post flair	Combined engagement, mean (SD)	Score, mean (SD)	Comments, mean (SD)
Pregnancy	6.16 (9.18)	1.93 (4.34)	4.23 (5.9)
Wound care	4.60 (9.12)	1.74 (5.53)	2.86 (4.59)
Injury	4.59 (9.97)	1.88 (5.86)	2.71 (5.1)
Parasite concern	4.36 (6.31)	1.51 (2.93)	2.85 (4.23)
Skin issues, rashes, freckles, or moles	4.17 (8.93)	1.60 (4.89)	2.57 (4.58)
Genitalia	4.05 (6.89)	1.42 (3.18)	2.63 (4.32)
Cardiac	3.86 (6.65)	1.34 (2.03)	2.53 (5.01)
Mouth, gums, throat, or cheeks	3.86 (8.13)	1.47 (3.98)	2.40 (4.62)
Illness	3.80 (6.67)	1.39 (2.89)	2.41 (4.46)
Eyes	3.76 (7.41)	1.48 (3.62)	2.28 (4.28)
Mental health	3.65 (5.25)	1.35 (2.66)	2.30 (3.33)
Medication	3.58 (5.09)	1.28 (2.71)	2.30 (3.06)
Digestion, stomach, or bowels	3.45 (4.73)	1.24 (1.97)	2.21 (3.34)
Bones, joints, or ligaments	3.13 (4.34)	1.25 (1.90)	1.88 (2.85)

### Engagement by Media Inclusion

**Figure 3:**
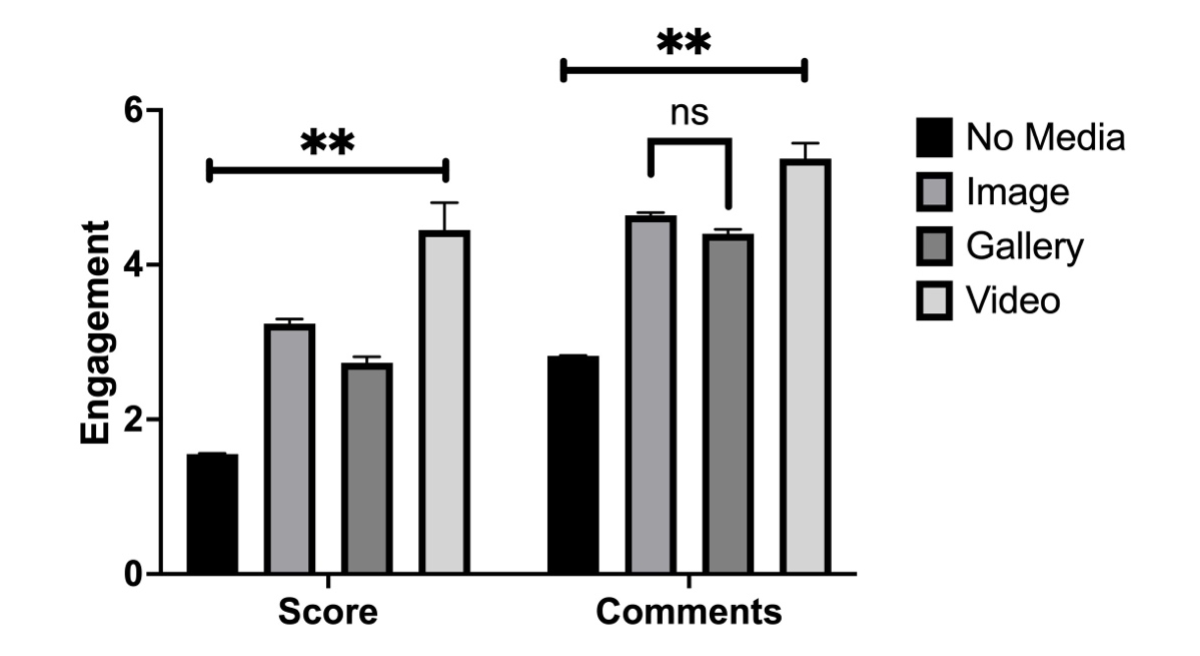
Engagement by inclusion of media. This bar graph illustrates the engagement of posts based on the type of media the posts include in r/medical_advice during all periods. Gallery means multiple images are included as part of the post. Error bars represent the SEM. Asterisks indicate the level of statistical significance (*P<.05 and **P<.01). ns: nonsignificant.

A total of 28% (56,533/201,904) of posts contained media in the form of images or videos. Of these, 20% (39,776/198,830) included a single image, 8% (15,149/189,363) included multiple images, 0.8% (1608) included a video, and 72% (145,147/201,000) did not include any media. Posts that included a single image received, on average, a score of 3.24 (SD 12.03) and 4.64 (SD 6.91) comments. Posts with multiple images received, on average, a score of 2.73 (SD 9.18) and 4.40 (SD 6.80) comments. Posts with a video received, on average, a score of 4.45 (SD 14.10) and 5.37 (SD 7.98) comments (Figure 3).

Posts with any media received, on average, a score of 3.14 (SD 11.41) and 4.60 (SD 6.92) comments, compared with a score 1.55 (SD 4.15) and 2.82 (SD 3.70) comments for posts without any media. Compared with posts without media, those with media received higher engagement (Dunn test; scores *P*<.001, comments *P*<.001). There was a significant difference between engagement of videos, multiple images, and a single image (Dunn test; scores *P*<.001; comments *P*<.001). Posts with videos received the highest engagement, followed by posts with images, and posts with no media received the least. Furthermore, posts with multiple images received lower scores (*P*<.001) but a greater number of comments (*P*<.001) compared with posts with a single image.

## Discussion

### Principal Findings

Our study provides an in-depth examination of user dynamics within the subreddit r/medical_advice, illuminating the intricacies of online health information–seeking behaviors. Our findings align with established medical literature on online medical information seeking. Online health forums have been shown to frequently serve as primary sources for addressing nonurgent and less severe medical concerns [8]. The high volume of posts on noncritical health issues suggests a common use of these platforms. It is reasonable to think that users are seeking preliminary advice, or perhaps just reassurance, before consulting a health care professional due to the ease of access to online medical information. Of note, the high level of engagement with pregnancy-related posts is a trend mirroring other online health communities [9], highlighting a consistent public interest in reproductive health.

In addition, our study explored the engagement dynamics of posts containing visual media, an area of study that is lacking in current medical literature. Our results show that posts featuring images or videos, especially concerning dermatological issues such as skin rashes or moles, have attracted higher levels of engagement. This observation not only underscores the effectiveness of visual aids in communicating complex medical information but also hints at a growing user preference for multimedia content [10]. With the rise of telemedicine and digital health communication in the post–COVID-19 era, the importance of visual aids in enhancing both diagnosis and patient understanding cannot be overstated.

Another intriguing aspect of our study is the significant contribution of nonverified medical professionals in providing advice. Our results show that r/medical_advice relies heavily on contributions from laypersons. This may be due to the lack of a robust verification process on the platform as it relies on the user to self-identify. This trend reflects a broader shift in the digital health information landscape, where community-based knowledge exchange is becoming increasingly predominant over traditional expert-driven models. While this democratization of health information has its advantages, it also inevitably raises concerns about the accuracy and reliability of the advice shared—challenges that have been extensively documented [11].

A key limitation of this study is that 41% of posts lacked user flairs, which leaves a significant portion of users’ backgrounds unclear. We acknowledge this as a potential source of bias and recommend future investigations using natural language processing or other linguistic analysis methods to characterize these flairless users, which could enhance our understanding of their information-seeking patterns. In addition, by focusing solely on a single subreddit, we acknowledge that our findings may not fully represent online health-seeking behaviors across various platforms and communities. The unique characteristics of r/medical_advice—including its user demographics, content moderation practices, and engagement patterns—may not perfectly mirror those of other online health forums. Furthermore, the study’s reliance on user-generated categorizations for post flairs and the self-identification of medical professionals introduces potential biases and inaccuracies, which could affect our interpretation of the data [12].

In terms of future directions, numerous opportunities for further research present themselves. Comparative studies across various social media platforms could examine unique trends and user behaviors, offering a more comprehensive picture of online health-seeking patterns. Further investigation into the truthfulness and impact of advice provided by online users remains a critical area of exploration [13]. In addition, understanding the motivations behind patients turning to social media for medical advice, and the consequences of acting on potentially incorrect information, is important to assess these platforms’ impact on public health and health care costs.

### Conclusion

Our investigation into r/medical_advice uncovers a complex and evolving landscape where online platforms serve as significant avenues for medical inquiry and information exchange. This study highlights the role of both professional and nonprofessional users in shaping these interactions and emphasizes the value they bring. While these platforms may offer invaluable opportunities for information sharing and support, the variable quality and reliability of the advice provided require careful consideration from the professional medical community. There is a clear need for increased participation from verified medical professionals and the implementation of effective moderation policies to ensure that online health forums function as reliable and supportive communities for individuals seeking medical guidance. Such measures are vital to mitigate the risks of misinformation and foster a safer, more informed online health ecosystem.
